# Factors influencing societal response of nanotechnology: an expert stakeholder analysis

**DOI:** 10.1007/s11051-012-0857-x

**Published:** 2012-05-01

**Authors:** Nidhi Gupta, Arnout R. H. Fischer, Ivo A. van der Lans, Lynn J. Frewer

**Affiliations:** 1 Marketing and Consumer Behaviour Group, Wageningen University, Building 201. Hollandseweg 1, 6706 KN Wageningen, The Netherlands; 2 School of Agriculture, Food and Rural Development, Newcastle University, Agriculture Building, Newcastle upon Tyne, NE1 7RU UK

**Keywords:** Nanotechnology, Societal response, Expert opinion, Factors, Applications of nanotechnology, Repertory grid method, Generalized Procrustes analysis, Societal implications

## Abstract

Nanotechnology can be described as an emerging technology and, as has been the case with other emerging technologies such as genetic modification, different socio-psychological factors will potentially influence societal responses to its development and application. These factors will play an important role in how nanotechnology is developed and commercialised. This article aims to identify expert opinion on factors influencing societal response to applications of nanotechnology. Structured interviews with experts on nanotechnology from North West Europe were conducted using repertory grid methodology in conjunction with generalized Procrustes analysis to examine the psychological constructs underlying societal uptake of 15 key applications of nanotechnology drawn from different areas (e.g. medicine, agriculture and environment, chemical, food, military, sports, and cosmetics). Based on expert judgement, the main factors influencing societal response to different applications of nanotechnology will be the extent to which applications are perceived to be beneficial, useful, and necessary, and how 'real' and physically close to the end-user these applications are perceived to be by the public.

**Electronic supplementary material:**

The online version of this article (doi:10.1007/s11051-012-0857-x) contains supplementary material, which is available to authorized users.

## Introduction

Emerging applications of nanotechnology have the potential to deliver new manufacturing processes and products across various different sectors of application, ranging from agriculture to medicine to defence applications, which will potentially result in profound changes in society as a whole (Crow and Sarewitz 2001). To realise the full potential of nanotechnology, significant resources have been allocated for nanotechnology research by government institutions, public and private research centres, universities and industry globally (Brossard et al. 2009; Roco 2003; Roco and Bainbridge 2005; Salerno et al. 2008). However, the potential social and economic benefits of nanotechnology may not be realised if the issue of societal acceptance of nanotechnology and the concrete products of its application, across a range of application domains, is not adequately addressed. In the past, societal responses to new technologies have played a crucial role in the success (e.g. mobile phones, internet) or failure (e.g. food irradiation; genetically modified foods in Europe) of such technologies (Frewer et al. 2004, 2011a; Gaskell et al.^1999^; Van Kleef et al. 2010; Wright and Androuchko 1996). It is likely that, just as has been the case for some other new technologies, socio-psychological factors will influence the societal response to nanotechnology (Gupta et al. 2011). It is recognised that such socio-psychological factors will shape the commercialisation trajectory of technology, but also facilitate allocation of resources in areas of application relevant to the wider needs of society. Thus, the identification of these factors will play an important role in the future development of nanotechnology.

From the literature, there is some evidence that, at the present time, the general public has limited, or no, knowledge or awareness about nanotechnology, and that public involvement in the debate surrounding nanotechnology development is rare (Pidgeon et al. 2009; Priest 2006; Ronteltap et al. 2011; Satterfield et al. 2009; Siegrist et al. 2008; Vandermoere et al. 2010). Therefore, at this stage in the development of nanotechnology, people with occupation related experience and expertise in nanotechnology from the scientific community, industry, policy makers or consumer representatives are likely to inform the development and application of nanotechnology.

An important element in determining how the technology will be implemented depends on the perceptions of these experts regarding societal acceptance of both the technology and its specific products across different domains of application. Although expert view on societal response to new technologies may not align with actual societal attitudes, (Barke and Jenkins-Smith 1993; Blok et al. 2008; Flynn et al. 1993; Kraus et al. 1992; Sjöberg 1999; Webster et al. 2010), those expert views on societal responses, are likely to influence technology implementation and commercialisation. Identifying expert priorities and preferences at an early stage of technological development can be used to identify how such views have influence on the commercialisation trajectory in the future. A study of these expert groups can provide an opportunity to examine which perceptions currently represent broadly shared consensus among the different stakeholder groups, and which are associated with a broader range of individual opinions (Besley et al. 2008). In addition research on expert views can provide a benchmark to analyse preferences and concerns, and may be used as a precursor to initiate dialogues at improving the practicality of regulatory actions (Berube et al. 2011). The present study can contribute to making future comparisons between public and expert views on societal issues related to nanotechnology as identification of the critical differences between expert and public opinion needs to be taken into account in framing risk communication efforts directed at public (Hagemann and Scholderer 2009).

The aim of this paper is to elicit expert opinion on factors influencing societal response to applications of nanotechnology. The specific objective of this study is to compare different applications of nanotechnology and identify expert views regarding factors influencing societal acceptability.

There have been some studies highlighting expert views on nanotechnology (Besley et al. 2008; Berube et al. 2011; Corley et al. 2009; Ho et al. 2011; Priest et al. 2010; Siegrist et al. 2007a; Yawson and Kuzma 2010). Yawson and Kuzma (2010), showed that according to experts factors such as trust, institutions, risk and benefit perception and knowledge are likely to affect consumer acceptance of agrifood nanotechnology products. Siegrist et al. (2007a) used the psychometric paradigm to examine risk perception and the role of trust in developing attitudes toward nanotechnology among laypeople and experts. This study suggested that perceived dreadfulness of applications and trust in governmental agencies are important factors in determining risks. It also emphasised that for an expert sample in the study, confidence in governmental agencies was an important predictor of risks associated with nanotechnology. Another study by Priest et al. (2010) compared the risk and benefit perception of nanotechnology among US citizens and a group of nanotechnology experts. The study showed that public opinion has started to diverge from expert opinion with respect to societal risks of nanotechnology as for citizens, there has been a rapid rise in concern over societal risks in comparison to risk associated with health and environment. A study on expert opinion on nanotechnology by Besley et al. (2008) showed that public health and environmental issues are the areas where both risk and need for regulation are greatest. Also while considering risk and regulation, experts distinguished between health, environment and social risks. U.S. nano-scientist’s risk and benefit perception of nanotechnology, as well as their support for nanotechnology regulation, showed that nano-scientists are more supportive of regulating nanotechnology when they perceive higher levels of risks; however, perceived benefits about nanotechnology do not significantly impact their support for nanotechnology regulation (Corley et al. 2009). Compared with the experts, the public judged nanotechnology as having greater risks and fewer benefits, and indicated less support for governmental funding of nanotechnology research (Ho et al. 2011).

Most previous research in this area has used a priori defined constructs, developed either from existing theoretical models which did not account for any specific concerns associated with public acceptance of the technology, or were decided by the researchers. To fully capture the factors that determine expert views on the societal response to nanotechnology, it would be advantageous not to make a priori assumptions about what expert consider to be important issues for societal acceptance (Frewer et al. 1997). Constructs elicited this way are likely to provide a more meaningful reflection of the real attitudes and perceptions of the group of participants being sampled (Henson et al. 2008). This, in turn, would help in evolving a more realistic picture of the potential factors driving societal response to nanotechnology and its applications. Repertory grid methodology in conjunction with generalized Procrustes analysis (GPA) offers a methodological solution. The repertory grid method (RGM) allows respondents to describe their response in their own words without imposing external, experimenter determined factors, while GPA allows the differentiation of constructs about which respondents agree, and the most important determinants can be identified (Frewer et al. 1997).

Elicitation of constructs is a complicated exercise, as too little structure makes the elicitation unfocused, while too much structure, unacceptably, limits the depth of the results. Some structure can be provided by discussing specific applications of nanotechnology, instead of the technology as a whole. Until now, research on public perception of nanotechnology has largely focused on nanotechnology in general rather than specific applications (Cobb and Macoubrie 2004; Gaskell et al. 2004; Lee et al. 2005; Scheufele and Lewenstein 2005), with the exception of few studies (Besley et al. 2008; Scheufele et al. 2007; Siegrist et al. 2007a, b; Stampfli et al. 2010; Yawson and Kuzma 2010). Previous research has shown that the public perception of new technologies depends on the type of application domain as well as specific application attributes (Bauer 2005; Frewer et al. 1997), emphasising the need to examine specific applications of nanotechnology within and between application domains (Pidgeon et al. 2009; Siegrist et al. 2007b).

To elicit constructs based on several different applications, the RGM combined with generalised Procrustes analysis, provides structure and the basis for systematic comparative analysis on the one hand, while simultaneously allowing the elicitation of the required depth of arguments on the other. The RGM originated in psychology, and has been used in number of consumer research studies across different disciplines (such as medicine, health and food) to elicit individual’s perception (Frewer et al. 1996, 1997; Lewith and Chan 2002; Messina et al. 2008; Mireaux et al. 2007; Rowe et al. 2005; Russell and Cox 2004; Tio et al. 2007). It can be used as a tool to facilitate a stakeholder dialogue on a societal issue (van de Kerkhof et al. 2009) and is particularly useful in consumer research in the early stages of product development (van Kleef et al. 2005). Advantages of using this particular method are: (1) It offers a structured method in exploring individual perceptions without imposing researcher bias or vocabulary (Mireaux et al. 2007; Schaffalitzky et al. 2009). (2) The method is efficient in identifying the full range of constructs that people use for evaluating an issue in a particular context with as few as 15 interviews (van de Kerkhof et al. 2009).

The data obtained using RGM can be analysed using generalised Procrustes analysis (GPA; Gower 1975), a multivariate statistical technique that aims to identify consensus between observer assessment patterns and provide a measure of observer agreement with as little intervention of the researcher as possible (Wemelsfelder et al. 2000). By analyzing the results using GPA, variations due to assessors using different terms to describe the same stimuli and/or variation in their use of rating scales can be controlled (Mireaux et al. 2007).

## Methods

Structured interviews with experts on nanotechnology from North West Europe were conducted using repertory grid methodology.

A list containing a broad range of different applications of nanotechnology was prepared. In order to maximise chances of finding relevant dimensions, the applications of nanotechnology were selected from different domains (cf. Siegrist et al. 2007a). Following discussions with scientists directly involved in developing nanotechnology applications, the list was further developed and a final selection of 15 key applications of nanotechnology drawn from different areas (e.g. medicine, agriculture and environment, chemical, food, military, sports and cosmetics) was made. These 15 applications of nanotechnology were then used to elicit the underlying constructs. A list of these applications is provided in Table 1.

**Table 1:** Specific applications used in the generation of constructs about nanotechnology

1. Targeted drug delivery by medically functionalized nanoparticles
2. Neuro-implantable devices designed using carbon nanotubes used for simulating brain circuit activity
3. Easy to clean surfaces made using nanomaterials, e.g. self-cleaning windows
4. High-volume manufacture of very inexpensive RFID tags using nanoparticles
5. Encapsulation and delivery of nutrients in food using nanomaterials
6. Food packaging using nanoparticles with antimicrobial properties to increase shelf life of food products
7. Smart pesticides developed using nanotechnology to enhance the effectiveness or delivery of pesticides
8. Chemical sensors designed using nanomaterials (such as carbon nanotubes, zinc oxide or nanowires) to detect very small amounts of chemical vapours
9. Membranes made of nanomaterials to build light weight and longer lasting fuel cells
10. Remediation of contaminated water or soil using nanoparticles
11. Development of efficient and cost effective water filtration processes by using nanomaterials (carbon nanotubes and nanoparticles)
12. Smart dust designed using nanotechnology for tracking changes in environment used in military intelligence
13. Cosmetics containing nanoparticles used to enhance active ingredient absorption (e.g. sunscreens; anti-ageing creams), and facilitate repair damage (combat hair loss, prevent greying hair)
14. Nanofabrication to get desired properties in the fabric such as making them antimicrobial, water and stain resistant, fire resistant or bulletproof
15. Sturdy and better quality sports goods designed using nanomaterials e.g. golf clubs, tennis rackets, balls etc.

### Participants

A range of experts from North West Europe, who were engaged in diverse activities related to nanotechnology, were recruited into this study. An initial list of potential participants was compiled using the networks of the authors (Frewer et al. 2011b). In addition, the names of potential participants were also compiled from open sources such as the list of participants from a conference on nanotechnology, and the authors of publications related to nanotechnology. From the initial list, a cross section of experts across the key stakeholder groups of academia, industry, government, media and consumer representative groups was invited to participate. Snowballing by asking participants to identify additional experts was used to complete the list. The response rate was 90 %, resulting in 18 experts who agreed to take part in the study. One participant showed unwillingness to follow the protocol and the data they provided was not further analysed, leaving 17 valid responses, 15 men and 2 women^1^ (mean age = 50.7 years, SD = ± 7.1 years) across all stakeholder groups (Table 2).

**Table 2:** Expert groups

Expert affiliation	Specific professional field
Academia	1. Biochemistry and Toxicology
	2. Environment and Agriculture
	3. Risk perception and Communication
	4. Polymer Technology
	5. Material Science
	6. Chemical Sensors
Industry	7. Medical
	8. Food
	9. Water Filtration
	10. Cosmetics
	11. Polymer/Fabrics
Government/regulatory authorities	12. Ministry of Agriculture
	13. Ministry of Defence
	14. European Commission
	15. Food Safety Authority
Consumer representative group	16. Consumers and Nanotechnology
Media	17. Biotechnology Journalism

### Design

The set of 15 applications was developed and refined in discussion with nanotechnology experts from the host institution of the authors. The survey used 10 triads compiled from a set of 15 specific applications of nanotechnology to start the elicitation of expert’s opinion. Triads were presented in randomised order with each application being presented twice (in different triads) to each participant. For each triad, participants were asked, ‘which 2 out of these applications of nanotechnology do you find to be similar in terms of societal response, and why?' and ‘which of these application of nanotechnology is different from the other 2 applications in terms of societal response, and why?' to create bipolar arguments on differences between the applications. Once all 10 triads had been used to elicit arguments for societal response, or when no new arguments were elicited following presentation of 3 consecutive triads, experts scored each of the applications of nanotechnology on each of the arguments on a 5-point scale with personalised labelled end points derived from elicitation. Out of 17 participants, one participant could only use 9 triads to elicit arguments for societal response. The interview was prepared and piloted with 3 experts from the host institution, after which adjustments were made.

### Procedure and data-collection

The data were collected in a face-to-face interview. The interview was divided into 2 phases. In the first phase, constructs describing determinants of societal response to nanotechnology were elicited, after which a small break was suggested. This was followed up by the second phase where the experts rated each of the applications on each construct they had personally described as relevant. Interviews were conducted using Idiogrid software (Grice 2002). The interviews with experts were conducted between October 2010 to April 2011. Interviews were audio-taped after receiving verbal consent from the interviewee to allow more in-depth interpretation of expert opinions. On average it took 50 min to complete the interview. Interviewees received a token gift (worth about 10 Euro) as appreciation for their time.

### Analysis

The aggregated data from the 17 experts consisted of 338 constructs in total. The number of constructs elicited from each expert ranged from 14 to 20, with the mean number of constructs being 18.2. The first author classified the constructs into series of construct- classes. Subsequently, the second author applied the initially defined construct classes to the constructs after which modifications were made to the construct classes. A final check of the emerging classification scheme was conducted by the last author, who had not been involved in the classification until that time. When disagreement occurred, the classification was discussed until agreement was reached. The construct classes were based on abstractions of the actual constructs; for example, if an expert stated that he or she found the applications ‘helpful for more people', this was deemed to fall within the class of ‘larger societal benefits'. Some constructs were classified as combination of two construct classes for e.g. ‘human health benefits + personal benefits'. This process resulted in 58 construct classes.

In order to check the classification conducted by the authors, another member from the host institute, who was not involved in the research, was asked to conduct an independent coding of the constructs given by the experts, using the 58 construct classes defined by the authors. A Cohen’s kappa of 0.79 indicated very good agreement between the coders regarding the classification of the constructs. Differences were then resolved by further discussion to achieve consensus on classification and in total 57 construct classes were retained. Details of the constructs elicited by experts and the construct classes assigned are provided in Online Resource 1. The classified data were then analysed using GPA (Gower 1975) and further interpretation was done using principal component analysis (PCA).

All the 17 grids from the experts were analysed using GPA. GPA considers each grid as a multidimensional geometric configuration, taking an expert’s (classified) constructs as dimensions and the scores that the expert gave on these for each application as coordinates for the different applications. Each configuration has as many dimensions as it has constructs, and the 15 applications of nanotechnology are represented as points in this multidimensional space. The 17 configurations thus obtained are then matched to each other through a series of iterative mathematical transformations (rotation/reflection and scaling), while preserving inter-sample relationships within each configuration (Wemelsfelder et al. 2000). After convergence of the iterations, a ‘consensus grid’ is calculated by taking the mean of all transformed individual configurations. The match between the transformed individual grids and the consensus grid is expressed in terms of the ‘consensus proportion', providing the proportion of variance in the individual’s grids that is accounted for by the consensus grid (similar to *R* ^2^ in ordinary least squares regression analyses). It should be kept in mind however, that a perfect match (consensus proportion equal to 1) only implies that individual configurations can be aligned, but not necessarily that experts used the same constructs, nor that they rated the applications in the same way on similar constructs. That is, the obtained consensus grid is entirely independent of interpretive judgement by the researcher, is defined purely in terms of its geometrical properties, and has no semantic connotations attached to it. The match among individual grids in terms of semantic connotations will be assessed while interpreting more detailed results. The consensus proportion was tested for statistical significance using a randomisation test (Wakeling et al. 1992). In order to make further interpretation, the consensus grid was submitted to a PCA to extract the main dimensions (Grice and Assad 2009). Finally, the principal components (PC) are interpreted by inspecting their relation with the 338 classified constructs.

Once the main factors influencing societal response to nanotechnology using GPA and PCA were identified, the transcribed interviews were reviewed to identify statements that supported expert views on regarding these factors explaining differences in expert views associated with the different applications of nanotechnology.

## Results and interpretation

The consensus proportion was found to be 0.60 indicating that the GPA consensus grid represented experts’ judgements about the 15 applications with respect to their self-generated constructs fairly well. 1,000 trials were generated based on the current data and showed that the observed consensus proportion was indeed significant (*p* < 0.001). Consensus proportion for only one expert was found to be 0.21, while for all the other experts it ranged from 0.46 to 0.72, indicating that there was relatively little variance in response with respect to the consensus grid. Consensus ratio’s for applications of nanotechnology ranged from 0.41 to 0.73 (Table 3). Higher consensus among expert views was found for applications like easy to clean surfaces, smart dust, encapsulation and delivery of nutrients in food, sports good, water filtration and medical applications of nanotechnology. More variation between experts opinion was found for applications such as nano fabric, fuel cells and food packaging there was more variation in expert’s opinion.

**Table 3:** Consensus proportion for applications of nanotechnology from lowest to highest consensus proportion

Application	Consensus/total
Nano fabric	0.41
Fuel cells	0.45
Food packaging	0.48
Chemical sensors	0.50
Smart pesticides	0.51
RFID tags	0.54
Cosmetics	0.55
Targeted drug delivery	0.61
Neuro-implantable devices	0.61
Water filtration	0.61
Soil water remediation	0.63
Sports goods	0.63
Encapsulation and delivery of nutrients in food	0.65
Smart dust	0.72
Easy to clean surfaces	0.73

The consensus grid obtained through GPA was subjected to PCA with promax rotation. Examination of the scree plot suggests confining the interpretation of results to four PC with the first six eigenvalues of the unrotated components being 3.92, 2.19, 1.62, 1.00, 0.39 and 0.24, explaining 87.3 % of the total variance.

**Fig. 1:**
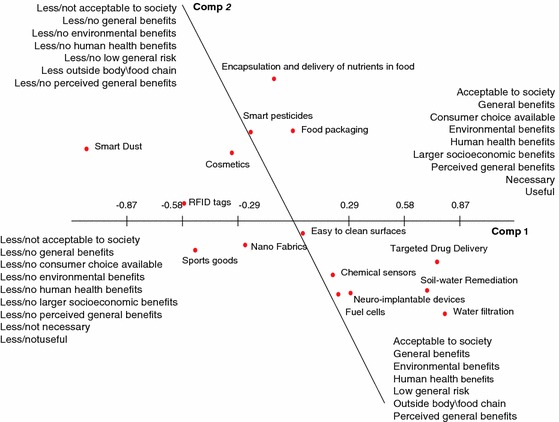
Location of applications of nanotechnology on first and second principal component

**Fig. 2:**
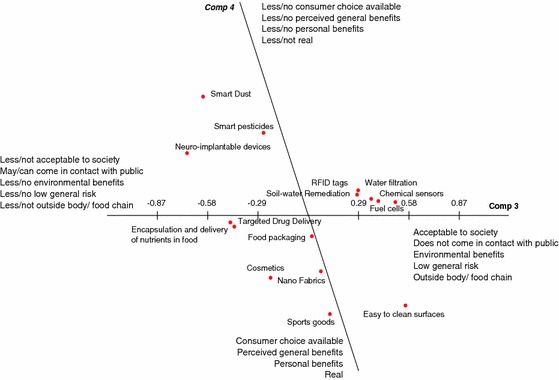
Location of applications of nanotechnology on third and fourth principal component

To interpret these four PC (labelled PC1 through PC4) the structure loadings of each construct was calculated for each respondent. To summarise these loadings of 338 constructs on 4 components, a count was done for the number of high loadings (≤−0.50 or ≥0.50) for each construct class on each principal component. Construct classes that have at least 3 times a high loading on a component were deemed important for the interpretation of that component (highlighted in bold in Table 4). In addition, Figs. 1 and 2 give plots of these loadings, providing the contours of the four main dimensions of the consensus grid that describes how experts as a whole perceived 15 applications of nanotechnology in terms of societal response.

**Table 4:** Total number of constructs in each construct-class and the number of constructs with a high loading on the first four principal components (PC)

Construct class	PC 1 (33.05 %)		PC 2 (30.36 %)		PC 3 (25.55 %)		PC 4 (19.49 %)		Total
	(+)	(−)	(+)	(−)	(+)	(−)	(+)	(−)	
Acceptable to society	**3**	0	0	**4**	**6**	0	0	2.5	15.5
Benefits for a subgroup of people in society	1	0	0	1	1	0	0	0	3
General benefits	**5.5**	0	0	**4.5**	2	0	1	2.5	15.5
Comes into contact with public	1	0	0	0	0	1	0	0.5	2.5
Consumer choice available	**3**	0	0	0	0	0	0	**4**	7
Developing country benefits	1	0	0	0	0	0	0	0	1
Does not come in contact with public	0	1	0	1	**4**	0	1	0	7
Easy to sell	1	0	0	0.5	0	0	0	0.5	2
Easy to understand	0	0	0	0	0	0	0	1	1
Environmental benefits	**5**	0	0.5	**5**	**7**	0	0	2.5	20
Ethical issues	1	0	0	0	0	0	0	0	1
Fiction	0	0	0	0	0	1	2	0	3
Human health benefits	**14.5**	0	0	**7.5**	1.5	0	**3**	2.5	29
Larger socioeconomic benefits	**6**	0	0	2	1	0	0	1	10
Less acceptable to society	0	0	0	0	0	1	0	0	1
Low general risk	0.5	0	0	**3**	**3.5**	0	0	2	9
Necessary	**7**	0	0	2	0	0	0	1	10
“Nice to have” applications	0	1	1	1	0	0	0	2	5
No concern	0	0	0	2	1	0	0	1	4
No environmental risk	0	2	0	1	1	0	0	0	4
No ethical issues	0	0	0	1	0	0	0	0	1
No health risk	1	0	0	0	0	0	0	0	1
No perceived risk	0	0	0	0	0.5	0	0	0.5	1
Not novel\no value addition	0	0	0	0	2	0	0	2	4
Not of immediate interest	0	0	0	0	1	0	0	0	1
Not scary	0	0	0	0	1.5	0	0	0	1.5
Novel application\value addition	2.5	0	0	0	0.5	0	0	0	3
Outside body\food chain	0	1	0	**3**	**6.5**	0	0	0.5	11
Perceived general benefits	**5**	0	0.5	**4**	1	0.5	0	**7**	18
Perceived general risk	0	0	0.5	0	0	0.5	0	0	1
Personal benefits	0	0	0.5	1.5	0	1	0	**4**	7
Process oriented	1	0	0	0	0	0	0	0	1
Real	0	0	0	0	0	0	0	**3**	3
Useful	**3**	0	0	2	2	1	1	2	11

Construct class coded as a combination of two different construct classes was added as 0.5 to each of the classes separately allowing for decimals in the frequency count

The constructs used to describe determinants of societal response to different applications of nanotechnology on the positive end of PC1 are ‘*acceptable to society*', ‘*environmental benefits*', ‘*general benefits'*, ‘*perceived general benefits*', ‘*human health benefits*', ‘*larger socioeconomic benefits*', ‘*consumer choice available*', ‘*necessary*' and ‘*useful*’. Out of these construct classes, ‘*larger socioeconomic benefits*', ‘necessary' and ‘useful' are found to load only on PC1. This suggests that the first component is associated with the applications that are ‘**beneficial, useful and necessary**'. The negative end of PC2 is associated with the constructs ‘*general benefits*', ‘*environmental benefits*', ‘*human health benefits*', ‘*low general risk*', ‘*outside body\food chain*', ‘*perceived general benefits*' and ‘*acceptable to society*'. Unlike PC1, PC2 has no unique construct class, and is mainly found to address benefits and is therefore labelled as ‘**beneficial**'. The third principal component (PC3) relates to ‘*acceptable to society*', ‘*does not come in contact with public*', ‘*environmental benefits*', ‘*low general risk*' and ‘*outside body/food chain*'. Of these construct classes ‘*does not come in contact with public*' is only found to load on PC3. Hence, this component is primarily associated with ‘***distance from end user'***. Finally, the fourth principal component (PC4) has its negative extreme associated with ‘*consumer choice available*', ‘*perceived general benefits*', ‘*personal benefits*' and ‘*real*', while the only construct found to load on its positive end was “*human health benefits*”. The 2 construct classes that load exclusively on PC4 are ‘*personal benefits*' and ‘*real*', therefore the fourth component can be characterised as applications that are ‘***real and personal benefits***’.

A number of construct classes were found that loaded on more than one principal component. This can be interpreted by taking into account the correlation between the components. That is, if two components are correlated then an association between a construct classes and one of these two components is likely to also imply a correlation between the construct classes and the other component. As a high correlation is found between PC1 and PC2 (*r* = −0.51), most of the constructs that load on PC1 also load on PC2. There is no correlation between PC1 and PC3, and between PC1 and PC4. Similarly, there is no correlation between PC2 and PC3. There is moderate correlation found between PC3 and PC4 (*r* = −0.32), but they do not share any construct class that has many high loadings on both these components.

If a construct class has high loading on two different uncorrelated dimensions, this likely means that the construct class was used differently across experts. Construct class for example ‘acceptable to society' is found to load on PC1, PC2 and PC3. The loadings on PC1 and PC2 can be interpreted as similar as they are highly correlated components (applications that are beneficial, useful and necessary will be acceptable to society). The interpretation for the uncorrelated PC1 and PC3 of the construct class ‘acceptable to society' will be different as for PC1 acceptability to society seems to be used from the viewpoint of being beneficial, useful and necessary while for PC3 acceptability to society seems to be used from the viewpoint of not coming in direct contact with the public.

On the basis of the constructs associated with each principal component, it is possible to make some inferences about how experts have characterised the 15 applications of nanotechnology. Along PC1 (Fig. 1), which is primarily associated with beneficial, useful and necessary—low/no benefits, low/no usefulness and less/not necessary continuum, applications such as targeted drug delivery, neuroimplantable devices, water filtration, soil–water remediation, chemical sensors, and fuel cells are positioned positively, indicating these applications to be associated with higher benefits and are deemed necessary and useful, as a consequence, will be more acceptable to society:

[The] *public would only* accept new things *if they really benefited from [them]* (Industry, The Netherlands)

Applications such as smart pesticides, smart dust, RFID tags, nanofabrics, cosmetics and sports goods were rated less positively on this continuum, indicating these applications to be rated by experts as being perceived by society as less beneficial, less useful and less necessary:

*You do not need nanoparticles in cosmetics and food packaging* (Academia, The Netherlands)

Sports goods are *nice to have but not necessary* (Consumer representative group, UK)

Three applications were rated as neutral along this continuum. These were food packaging, encapsulation and delivery of nutrients in food and easy to clean surfaces. For these applications no clear consensus emerged in terms of benefits, usefulness, necessity and acceptability, for example an expert from industry explained:

*Research on Nano encapsulation is midway, if people feel it is all safe after safety evaluation, and people see that it has direct benefits for them, then they might accept them* (Industry, Belgium)

*They [encapsulation and delivery of nutrients in food] have to be genuinely useful for people to accept such things* (Consumer representative group, UK)

Of all the applications, smart dust was seen as unnecessary and least beneficial. The experts viewed targeted drug delivery and water filtration as the most beneficial and necessary applications of nanotechnology. All 17 experts agreed that targeted drug delivery was the most beneficial and necessary application of nanotechnology, and therefore will be the most societally acceptable application:

[The] *tendency of society is to accept medical applications more easily than other applications* (Government/regulatory authorities, Belgium)

Water filtration, on the other hand, was seen as necessary and beneficial in particular in the context of developing countries:

*Fresh drinking water will be very difficult in third world countries and at that point we need such applications* (Government/regulatory authorities, The Netherlands)

These application score similarly on PC2 that differentiates applications those are beneficial from applications that are less or not at all beneficial (Fig. 1). On this high benefit- to low benefits continuum, applications such as smart pesticide, smart dust, food packaging, encapsulation and delivery of nutrients in food, RFID tags and cosmetics are positioned on the positive side, indicating that they are associated with fewer benefits and more risks, for example:

*People don’t think about nanoparticles when it is in their [tennis] rackets and sports equipment, but they start to think of risks if these particles are in food* (Industry, The Netherlands)

*For pesticides, I always use the same analogy with recombinant DNA technology*—*no benefits to consumers, only benefit[s] to producers; there it has no chance of better acceptance in the society* (Industry, The Netherlands)

On the negative side of PC2 are the applications such as targeted drug delivery, water filtration, soil–water remediation, chemical sensors and fuel cells. These applications were seen as more beneficial with low risk, for example a governmental expert commented:

*Targeted drug delivery will bring direct benefits to the society* (Government/regulatory authorities, Ireland)

Applications that remain neutral on this scale are nanofabrics, sports goods and easy to clean surfaces. Water filtration was rated as being the most beneficial. Smart dust was considered to be the least beneficial of all applications of nanotechnology:

*Smart dust is like you have sensors all around you, it is not at all positive* (Industry, The Netherlands)

PC3 (Fig. 2), corresponds to the distinction between applications that come in contact with public, little risky and less acceptable to applications that do not come in contact with public and therefore more acceptable, for example:

[The] *closer it gets inside the body, [the] more resistant people would become [to] it* (Government/regulatory authorities, The Netherlands)

*In the beginning, to introduce the technology, it is better to start with membranes that do not come in contact with the public*—*first show everything is working without any problem* (Industry, The Netherlands)

Applications located on the positive side of PC3 are RFID tags, soil–water remediation, water filtration, chemical sensors, fuels cells and easy to clean surfaces. These applications are considered as being more distant from end-users.

*People will be able to see benefits in easy to clean surfaces as they [free nanoparticles] will not come in contact with the body* (Academia, UK)

On the negative side of this dimension were applications such as smart dust, neuroimplantable devices, smart pesticides, encapsulation and delivery of nutrients in food, cosmetics and targeted drug delivery, which are described as less distant from the end user.

*If smart pesticides enter the body they have more chances of crossing over the cellular barriers and reach somewhere in the body that the conventional pesticides couldn’t have reached* (Academia, UK)

Food packaging, nanofabrics and sports good were rated as neutral applications on this continuum on PC3.

Finally, PC4 (Fig. 2), differentiates between applications that are real and accrue personal benefits to the public from applications that appear less real with no/less personal benefits. Smart pesticide, smart dust and neuroimplantable devices are seen as less real, for example with regard to neuroimplantable devices an expert from industry commented:

*Neuroimplantable devices can be manipulated; it’s kind of scary for people, something like a science fiction* (Industry, Germany)

Experts rated sports goods, easy to clean surfaces, nanofabrics and cosmetics as applications that are more real, with more personal benefits. The remaining applications were ranked as neutral along PC4. These were RFID tags, soil water remediation, water filtration, chemical sensors, fuel cells, targeted drug delivery, food packaging and encapsulation and delivery of nutrients in food:

*RFID tags have slightly more distant business benefits, people might not care about it* (Media, Germany)

## Discussion

The present study investigated the views of the expert community regarding the potential societal responses to different applications of nanotechnology. Based on expert judgement, main factors influencing societal response to different applications of nanotechnology will be benefits, usefulness, necessity, issue of how close is an application from the end user, and how real these applications seem to be for them.

Benefits were generally mentioned by the experts included in this study before risk perception when discussing societal response. Risk perception is mainly mentioned as the opposite of benefit, rather than as a primary evaluative dimension. The present study shows that, according to experts, benefits will be the dominant factor that people would consider while making their choice for nano-products. Despite evidence that some people believe nanotechnology is riskier than do experts, many studies on public opinion on nanotechnology show that the public believes that benefits of nanotechnology will outweigh the risks (Burri and Bellucci 2008; Priest and Greenhalgh 2011; Satterfield et al. 2009; Scheufele and Lewenstein 2005; Stampfli et al. 2010), in line with the perceptions of experts in the current study. In contrast, other researchers have emphasised that societal responses to nanotechnology are likely to focus on risk rather than benefits (e.g. Marchant et al. 2008; Ronteltap et al. 2011; Sheetz et al. 2005).

Medical application (targeted drug delivery) was rated as the most societally acceptable application of nanotechnology by experts. Application with environmental benefits such as water filtration, soil–water remediation, fuel cells and chemical sensors were seen by experts to be the most beneficial applications of nanotechnology and likely to be societally acceptable. Nanotechnological innovations specifically in medical and environment domains have identified public perceptions of benefit and optimism regarding successful implementation (Besley et al. 2008; Burri and Bellucci 2008; Priest and Greenhalgh 2011).

In addition, the concept of need and usefulness has emerged as important construct classes in the analysis. According to the expert community, public response to a particular application of nanotechnology will not just focus on perceived benefits alone but also emphasise on questions relating to whether that application is necessary or whether it is seen as ‘trivial' and whether an application is useful. For example, water filtration was seen as beneficial and the most necessary application, in particular in context to the developing countries. Similarly, targeted drug delivery was deemed necessary in treating illness. Applications such as sports goods and cosmetics were seen as ‘nice to have applications' but not necessary.

The notion of “distance from end user” also emerged as an important factor. According to experts, people will make their decisions about the acceptability of a particular use of technology by assessing the possibility of coming into contact with the nanomaterial, or the chances of migration of nanoparticles into the body or food chain. Therefore acceptability will not just depend on benefits but also be influenced by distance from end user. This particular factor may play an important role in shaping societal response to nanotechnology applications in the food domain; while the benefits associated with medical applications (for example, targeted drug delivery) are thought to be perceived to outweigh the risks. There is still no consensus on whether application of nanotechnology in food will be acceptable to society. For example, there is evidence to suggest that Swiss citizens were concerned about the migration of nanoparticles (Burri and Bellucci 2008), in the body or environment. The food domain has been reported to be perceived differently to other domains in the US (e.g. energy-related and medical applications). A close link between acceptability and risk–benefit judgements associated with perceived ‘bodily invasiveness' was also observed (Conti et al. 2011). Research conducted in Switzerland has demonstrated that people perceive nanotechnology food packaging as more beneficial than foods processed using nanotechnology (Siegrist et al. 2007b; Siegrist et al. 2008), while research done in France shows that people in France are pessimistic about both of these applications (Vandermoere et al. 2011). This highlights the need to take into account cross-cultural factors in determining acceptability, an issue not raised by the expert participants included in the study presented here.

The final factors that emerged from the analysis consist of notion of realism and personal benefits. Experts were of the opinion that people will distinguish between applications on the basis of the personal advantages that would accrue to an individual, and how real or close to reality these applications will appear to the public.

The use of RGM in conjunction with GPA facilitated the elicitation of a number of constructs by experts without imposing researcher bias. However, to interpret the broad range of elicited constructs, coding the responses into fewer construct classes is required. Although the methodologies have been developed to ensure a reliable coding scheme, this may have inadvertently introduced some researcher bias. An alternative approach may be to ask experts themselves to code the elicited constructs into construct classes which are agreed upon by the entire group of experts, using a Delphi-like process (Frewer et al. 2011b).

The methodology used in this study facilitates in identifying areas of consensus and similarities among the respondents. The differences among expert view could be either due to differences in opinion or due to uncertainties associated with the extent to which an individual expert is certain of the relevance of a particular construct to each application or application domain. These differences remain to be evaluated and future research is required in this direction. In addition, the present study provides a snapshot of expert opinions from North West Europe which may limit the geographical generalizability of the results. Future research should seek to compare responses of experts from different countries to present a complete overview on factors influencing societal response to nanotechnology. Finally, the elaboration of current research on ‘expert stakeholders' compared with the ‘lay public' is essential. The results presented here may contribute in making these future comparisons.

Fifteen out of 17 experts made direct comparisons between nanotechnology and genetic modification while discussing development of food applications and pesticides using nanotechnology. It has been noted that the experiences with genetically modified organisms and other controversial technologies have been linked with new technologies (including nanotechnology (Marchant et al. 2008; Frewer et al. 2011a). This suggests that experts speculate that social negativity will arise as nanotechnology is commercialised, in particular within the agrifood sector, and that at this stage in implementation understanding *why* this occurred with genetic modification may be useful when determining how nanotechnology might be commercialised.

Finally, the views of experts regarding the extent to which different applications of nanotechnology will be societally acceptable are likely to determine how and when these different applications are commercialised. Many experts in the study sample were of the opinion that the introduction of nanotechnology might follow the same course as that of genetically modified organisms, unless a more societally relevant innovation trajectory were adopted, and this might explain why participants emphasized the role of perceived benefit in terms of societal acceptance of nanotechnology applications. Assuming that experts shape the process of innovation, one might anticipate that the first products introduced into the (European) market will be those which experts perceive will be viewed as most beneficial and least related to societally less acceptable application in, for example, the agrifood sector. If this is indeed the case, the success of such an approach in terms of societal acceptance of specific nanotechnology applications can be evaluated, and contrasted to the case of genetically modified food where the applications initially introduced were not perceived to be beneficial by the public.

It is of interest that societal perceptions of risk are less often taken into account as primary evaluative dimension in expert analyses of the factors determining societal acceptance, and this may reflect an expert bias towards identifying an optimal commercialisation strategy rather than one focused on the application of precautionary regulation or other measures aimed at extremely low risk levels; regardless of potential benefits lost to society as a whole, or individual end-users.

In addition, consumer decision-making may be differentially biased by perceptions of risk, and this effect may further depend on the area of application for example, risks associated with nanotechnology and food production may be weighted more heavily than those associated with medicine when consumer decisions about acceptability are made. Further research is needed in this regard.

## Conclusions

The results of this study show that, according to nanotechnology experts, the general public will differentiate nanotechnology applications based on the extent to which they are beneficial, useful, necessary, real and to which the end-user is physically close with them. Risk is less often described by experts as a potential factor shaping societal acceptability. In part, this reflects expert opinions of how lessons from the commercialisation of genetic modification may inform market entry of products made through application of nanotechnology, and shape the associated commercialisation trajectory. It also reveals experts recognition that societal demand for concrete and necessary benefits will increase demand for specific products, and that a ‘consumer led' product development strategy is required. The lack of recognition of the primary role of perceived risk in societal decision-making suggests that stakeholders in commercialisation of nanotechnology may need to consider further how consumers make trade-offs between perceived risk and benefit, in particular in more controversial areas of application such as the agrifood sector.

## Electronic supplementary material

Below is the link to the electronic supplementary material.

Supplementary material 1 (PDF 220 kb)

## Footnotes

1 Although an effort was made to have a gender balance in the sample, more male respondents agreed to participate in the study.
