# Supplementary material for: Factors influencing societal response of nanotechnology: an expert stakeholder analysis
Source: J Nanopart Res. 2012 May 1;14(5):857. doi: 10.1007/s11051-012-0857-x (PMC3397131; doi:10.1007/s11051-012-0857-x)
Supplement: Supplementary file 1 — Supplementary material 1 (PDF 220 kb) [file 11051_2012_857_MOESM1_ESM.pdf]

Title: Factors influencing societal response of nanotechnology: An expert stakeholder analysis

Journal: Journal of Nanoparticle Research

Authors: Nidhi Gupta (Corresponding Author), Arnout R.H. Fischer, Ivo A. van der Lans and Lynn J. Frewer

Address of Corresponding Author: Marketing and Consumer Behaviour Group, Wageningen University. Building 201. Hollandseweg 1. 6706 KN Wageningen. The Netherlands. Email address: nidhi.gupta@wur.nl

	Constructs by Experts	Construct class		Constructs by Experts	Construct class
**PP #1**	**Similarity between 2 of the applications**			**Dissimilarity of the third to the other 2 of the applications**	
	perceived very well by public	perceived general benefits		could be perceived less well/it is not perceived at the moment	no perceived risk general+ no perceived benefits
	future technologies perceived necessary	necessary		not perceived necessary in Europe	non necessary
	to save the world	necessary		convenience products that are nice to have	nice to have applications
	perceived positively	perceived general benefits		negatively perceived	concern
	perceived most desired applications of nanotech	perceived general benefits		convenience product nice to have but can do without it	nice to have applications
	health benefit	human health benefits		Science fiction	fiction
	ethical dimensions are involved	ethical issues		basic technology	easy to understand
	realistic	real		fiction	fiction
	nice to have applications	nice to have applications		ethical and health implications	ethical issues + health risk
**PP #2**	industrial applications does not come in contact with public	do not come in contact with public		come in contact with public	come in contact with public
	possible positive effect	perceived general benefits		spying technology not positive at all	ethical issues
	potential benefit	perceived general benefits		could be used in a wrong way	could be misused\abused
	more primary needs for society	larger socioeconomic benefits		luxury good	nice to have applications
	we cannot do without these applications	necessary		we can do without these applications	non necessary
	nanoparticles in them do something positive	perceived general benefits		can be abused	could be misused\abused
	still choose if you want or not/results are positive	consumer choice		not desirable/coming too close to personal functioning	concern + ethical issues
	technology adds value to the environment	novel application\value addition + environmental benefits		technology can be misused	could be misused\abused
	life saving	human health benefits		non-essential product	non necessary
	lifesaving application	human health benefits		environmental friendly but not life saving	environmental benefits
**PP #3**	more risk benefits relationship	perceived general benefits + perceived risk general		people don't think about risks	no perceived risk general
	seen as more essential for daily life	necessary		goods that you can do without	non necessary
	added new functionality	novel application\value addition		application does not need any improvement by adding nanoparticles	non necessary
	improve health and environment	human health benefits + environmental benefits		less welcomed by public	less acceptable to society
	not interfering with personal life	do not come in contact with public		less favourable as its coming too close to people	come in contact with public
	direct improvement of one's personal life	personal benefits		do not have a direct link to personal use	no personal benefits
	Application beneficial for more people	larger socioeconomic benefits		Application beneficial for agriculture industry	benefits for a subgroup of people in society
	People see more direct benefit	perceived general benefits		people see lesser direct benefit	no perceived benefits
	nanotechnology improvises and makes the product cheaper	general benefits		Gives false sense of security may be the application is not efficient	perceived risk general
	reduces the use of chemicals	environmental benefits		making people afraid	scary
**PP #4**	not applied to skin or body	outside body\food chain		applied to the skin	inside body\food chain
	not easily acceptable depends on information	less acceptable to society		accept as long as people don't know about nanotech application	acceptable to society
	direct use for end user	useful		no direct use for end user	no personal benefits
	Consumer do not care if it is nanomaterial	not of immediate interest		consumer concerned about coming in contact with nanomaterials	concern+ come in contact with public
	related to life saving or threatening	human health benefits		add on application that you have anyway	not novel\no value addition
	working in the background	do not come in contact with public		day to day use	useful
	not scary	not scary		scary	scary
	nothing novel	not novel\no value addition		novel application\value addition	novel application\value addition
	helpful for more people	larger socioeconomic benefits		helpful for few people	benefits for a subgroup of people in society
	accessible to consumers	consumer choice		not accessible to consumers	no consumer choice
**PP #5**	non-invasive application in body	outside body\food chain		invasive application in body	inside body\food chain
	safe as nanoparticles are inside the matrix	low risk general		more toxicological risk	health risk
	Applied on surface	outside body\food chain		inside the body	inside body\food chain
	outside food chain	outside body\food chain		inside food chain	inside body\food chain
	no interaction with the nanoparticles with the environment	no environmental risk		nanoparticles may interact with the food	inside body\food chain + health risk
	preventive or curative to human health	human health benefits		technical application nothing to do with human enhancement	no human health benefits
	preventive measurement for many people	human health benefits		Curative for sick people	human health benefits
	improving the environment	environmental benefits		not improve the environment	no environmental benefits
	perceived non risky to human health	no health risk		perceived risk to human health	perceived health risk
	most desired and valued application	necessary		relatively less desired application	non necessary
**PP #6**	taken as consumer product making life easier	perceived general benefits		not used in usual households	not useful
	real applications	real		abstract and difficult to understand	difficult to understand
	perceived health benefits	human health benefits		scary	scary
	personal health and safety	human health benefits		more difficult to understand the concept	difficult to understand
	perceived as positive applications	perceived general benefits		Perceived as very negative application	concern
	broad application range for consumer	consumer choice		mass production in companies very narrow application range	process oriented
	promise to help third world countries	developing country benefits		more efficient mass production	process oriented
	catering to more broader consumers	consumer choice		more relevant for third world countries	developing country benefits
	save from pollution bringing in ecological benefit	environmental benefits		broad application range and benefit for consumer	consumer choice + personal benefits
	more desirable	necessary		hard to understand for laymen	difficult to understand
**PP#7**	outside body	outside body\food chain		inside body	inside body\food chain
	service for general public	larger socioeconomic benefits		means to go forward for patients	human health benefits
	cleaning environment	environmental benefits		carbon nanotubes may react with the environment	environmental risk
	outside the body so less concern	outside body\food chain + not scary		inside the body might raise concern	inside body\food chain + concern
	no fear using this application	not scary		fear associated with its use	scary
	protective and preventive	human health benefits		inside the body	inside body\food chain
	clear benefit to consumers\patients	personal benefits		not very clear benefit to consumers	no personal benefits
	More direct useful for people at large	larger socioeconomic benefits		more useful for environment conscious people	benefits for a subgroup of people in society
	preventing catastrophes	human health benefits		non preventive	no human health benefits
	closer to consumer in terms of contact and benefit	come in contact with public +benefits general		very far from consumer in terms of contact and benefit	do not come in contact with public + no personal
**PP#8**	necessary to prolong life	human health benefits		not necessary for living	non necessary
	more relevant function for protecting people	human health benefits		less relevant function	non necessary
	protective (health, environment and food security)	human health benefits + environmental benefits		not protective	no human health benefits
	use less debatable	acceptable to society		use is more debatable	less acceptable to society
	society will agree with the function of these applications	acceptable to society		society might not agree with the function	less acceptable to society
	solve medical problem where no alternative available	human health benefits		alternatives are available	not novel\no value addition
	more relevant to solve societal problem	larger socioeconomic benefits		you don't need nanotechnology and can avoid its function in this application	non necessary
	solve an existing problem	benefits general		does not solve any existing problem	no benefits general
	useful for daily purposes	useful		less useful for daily purposes	not useful
	can solve third world problems	developing country benefits		cannot solve third world problems	no benefits to developing countries
**PP#9**	there is no concern regarding leakage	no environmental risk		concerned about the leakage of particles	environmental risk
	helping to solve a problem	perceived general benefits		people don't see the advantages	no perceived benefits
	near term applications	real		more futuristic	fiction
	clear advantages to public	perceived general benefits		catering to military	benefits for a subgroup of people in society
	consumer issue	personal benefits		professional issue	benefits for a subgroup of people in society
	less concern about getting into the body	outside body\food chain		concern about bodily intake	concern +inside body

	Constructs by Experts	Construct class		Constructs by Experts	Construct class
	no privacy issue	no ethical issues		privacy issue and no advantage to consumer	ethical issues
	more easily acceptable/different position	acceptable to society		more difficult to imagine by the consumer	difficult to understand
	no concern	no concern		matter of concern	concern
	life saving	human health benefits		threatening having a wrong connotation	scary
**PP#10**	need driven application	necessary		luxury item	nice to have applications
	non luxury item	useful		luxury item	nice to have applications
	process oriented	process oriented		product oriented	product oriented
	deal with natural habitat/natural setting	no environmental risk		deal with modified habitat	environmental risk
	less difficult to accept	acceptable to society		difficult to accept	less acceptable to society
	more into the future	fiction		already existing	real
	aim to consumer	personal benefits		aims professional user	benefits for a subgroup of people in society
	biologically inclined	come in contact with public		non biologically inclined	do not come in contact with public
	utility oriented	useful		industry oriented	benefits for a subgroup of people in society
	more room for research	fiction		short term\current applications	real
**PP#11**	probability of generating least resistance from society	acceptable to society		fear of migration of particles in food	concern
	major benefits for people at large	larger socioeconomic benefits		benefit for limited number of people\particular section of people	benefits for a subgroup of people in society
	benefit for sustainable society	environmental benefits		marginal benefit to the society	few socioeconomic benefits
	major breakthrough for millions of handicap people	human health benefits		exist in early life and likely to bring some benefit	benefits general
	most likely to bring most benefit for the long term	benefits general		less of critical issue in long term trend	no concern
	increase sustainability agricultural productivity and enhance food production	environmental benefits		nice to have but not critical	nice to have applications
	minimal downside broader benefit	benefits general+ low risk general		overall balance of benefit and risks remains limited	perceived general benefits + perceived risk general
	both direct and indirect health and environment benefit	benefits for a subgroup of people in society		very limited in scope	benefits for a subgroup of people in society
	develop extremely powerful monitoring systems increasing efficiency of many things/range of application is infinite	benefits general		high marketing interest but low societal benefits	business benefits
	nice to have and some worthwhile (side) benefit	nice to have applications		nice to have but no real benefits	nice to have applications
**PP#12**	more acceptable outside the body	outside body\food chain + acceptable to society		people are more critical as inside the body	less acceptable to society + inside body
	no concern of nanoparticle leakage	no environmental risk		nanoparticles may enter the body	inside body\food chain
	do not have side effects on human health	no health risk		can have side effects on people health	health risk
	cleaning environment and food	human health benefits + environmental benefits		spying not acceptable	ethical issues
	better product less active component\renewability	environmental benefits		big border cannot see visibly	difficult to understand
	monitoring	do not come in contact with public		acting \doing something	come in contact with public
	people see them as useful application	useful		fear of misuse of application	could be misused\abused
	still some control of what you are doing	consumer choice		lack of knowledge	lack of knowledge
	more necessary	necessary		luxury item not really necessary	nice to have applications
	improvising health	human health benefits		not necessary	non necessary
**PP#13**	incremental enhancement similar to current use	not novel\no value addition		more unusual use of nanoparticles to get something new effect	novel application\value addition
	easy to understand by public	easy to understand		negative connotation	concern
	easy sell appearing low risk	easy to sell + low risk general		potentially ineffective and unsafe	not useful + perceived risk general
	low risk	low risk general		perceived unnatural	perceived unnatural
	higher appetite for risk	no concern		lower appetite for risk	concern
	unemotive industrial processes	no concern		hurdle to acceptability could be greater even though benefit could also be greater	less acceptable to society
	more acceptable due to underpinning regulatory safety net	acceptable to society		people might be concerned about the nanoparticles	concern
	potential for huge environmental and personal benefit	environmental benefits+ personal benefits		slightly more distant business benefit	benefits for a subgroup of people in society
	clear benefits to people	personal benefits		too spooky	scary
	consumer choice possible	consumer choice		consumer choice not possible	no consumer choice
**PP#14**	more environment benefits encouraging less use of chemicals	environmental benefits		concern about freedom of choice and privacy	no consumer choice +ethical issues
	can be seen having a utility to people	useful		issues with nanomaterials that are in contact with the body	come in contact with public
	Clear benefits	benefits general		suspicion about why these technologies are applied	could be misused\abused
	seen as nice to have	nice to have applications		emotive (negative) subject especially in EU	concern
	could have positive medical benefits	human health benefits		not necessary for modern society	non necessary
	potential medical benefits for anyone in the society	human health benefits		nice to have for certain consumer groups	nice to have applications
	contribute to sustainability and lower environmental footprint	environmental benefits		useful to have but more of a convenience	nice to have applications
	direct personal benefits	personal benefits		wider societal benefit	larger socioeconomic benefits
	people will be able to see its usefulness	useful		issue of whether its benefits for producer or public	benefits for a subgroup of people in society
	added value	novel application\value addition		non-essential	non necessary
**PP#15**	nanoparticles are not free to leave chances of exposure are lower	low risk general + no perceived risk general		chances of exposure to nanoparticles is higher	human health benefits + environmental benefits
	lots of benefit in terms of food safety	human health benefits		just recreational	nice to have applications
	health and wellbeing benefit	human health benefits		desirable	nice to have applications
	less risky and more beneficial	low risk general + benefits general		immediately raise lot of concern regarding access to unintended parts of the body	health risk
	not in contact with the body people do not perceive any risk	no perceived risk general+ outside body\food chain		come in contact with body so people might perceive slight risk	come in contact with public
	seen as useful in terms of health benefit	human health benefits		raise ethical and health issues	ethical issues+ health risk
	bring technological advantage to safety of environment	environmental benefits		misuse of technology	could be misused\abused
	relatively more acceptable because of greater benefit	acceptable to society + benefits general		uncertainty over fate behaviour and impact of nanoparticles	uncertainty
	more acceptable as particles are bound and do not come in contact with the body	acceptable to society + low risk general		suspicion of the safety of that particles can penetrate inside the skin	inside body\food chain + health risk
	relatively more acceptable	acceptable to society		perceived very risky	perceived risk general
**PP#16**	no entry of nanoparticles into human body	outside body\food chain		nanoparticles may come in contact with human body	inside body\food chain
	people accept easily as nanoparticles are bound in the matrix	acceptable to society +low risk general		people worried because of contamination of environment with nanoparticles	environmental risk
	public acceptance	acceptable to society		pubic opposition	less acceptable to society
	indirect application to society	do not come in contact with public		more concern about human contact	concern
	more acceptable	acceptable to society		relatively less acceptable	less acceptable to society
	novel application of nanotechnology	novel application\value addition		there are alternatives available no need of nanotechnology	non necessary
	less worry about nanoparticles	no concern		worry over contamination of food with nanoparticles	concern
	people accept it very easily	acceptable to society		people would need more information about its application to know its safe	lack of knowledge
	indirect process	do not come in contact with public		direct application chances of consuming nanoparticles	inside body\food chain
	encapsulated device	low risk general		consumer worry about possible effect on human health	concern
**PP#17**	people would see beneficial impact	perceived general benefits		general society would view it negatively	less acceptable
	direct benefit to society	larger socioeconomic benefits		interesting concept not directly applicable to general public	benefits for a subgroup of people in society
	desirability and necessity	necessary		not of immediate interest	not of immediate interest
	solving an ongoing problem	benefits general		not essential to everyday life	non necessary
	health priority attached to them	human health benefits		interesting but non-essential	nice to have applications + non necessary
	more societal benefit	larger socioeconomic benefits		less societal benefit	few socioeconomic benefits
	direct and immediate benefit to society	larger socioeconomic benefits		beneficial on a commercial scale	business benefits
	direct benefit	benefits general		negative connotations	concern
	easy sell to public	easy to sell		hard sell	hard to sell
	can provide improvement to food and environment safety	human health benefits + environmental benefits		luxury item	nice to have applications
